# Hepatic Fibrosis Progression in HIV-Hepatitis C Virus Co-Infection – The Effect of Sex on Risk of Significant Fibrosis Measured by Aspartate-to-Platelet Ratio Index

**DOI:** 10.1371/journal.pone.0129868

**Published:** 2015-06-19

**Authors:** Kathleen C. Rollet-Kurhajec, Erica E. M. Moodie, Sharon Walmsley, Curtis Cooper, Neora Pick, Marina B. Klein, Canadian Co-infection Cohort Study (CTN 222)

**Affiliations:** 1 Department of Medicine, Division of Infectious Diseases/Chronic Viral Illness Service, McGill University Health Centre, Montreal, Canada; 2 Department of Epidemiology & Biostatistics, McGill University, Montreal, Canada; 3 University Health Network, Toronto, Canada; 4 CIHR Canadian HIV Trials Network, Vancouver, Canada; 5 The Ottawa Hospital Research Institute, Ottawa, Canada; 6 Oak Tree Clinic, BC Women’s Hospital, Divisions of Infectious Diseases, Department of Medicine, University of British Columbia, Vancouver, Canada; University of Cincinnati College of Medicine, UNITED STATES

## Abstract

**Background:**

In Hepatitis C virus (HCV) mono-infection, male sex is associated with faster liver fibrosis progression but the effects of sex have not been well studied in HIV-HCV co-infected patients. We examined the influence of sex on progression to significant liver fibrosis in HIV-HCV co-infected adults receiving antiretroviral therapy (ART) using the aspartate aminotransferase-to-platelet ratio index (APRI) as a surrogate biomarker of liver fibrosis.

**Methods:**

We evaluated 308 HIV infected, HCV RNA positive participants of a Canadian multicentre prospective cohort receiving antiretrovirals and without significant liver fibrosis or end-stage liver disease at baseline. We used multivariate discrete-time proportional hazards models to assess the effect of sex on time to significant fibrosis (APRI≥1.5) adjusting for baseline age, alcohol use, cigarette smoking, HCV duration, and APRI and time-updated CD4 count and HIV RNA.

**Results:**

Overall, 55 (18%) participants developed an APRI ≥ 1.5 over 544 person-years of at-risk follow-up time; 18 (21%) women (incidence rate (IR)=14.0/100 PY; 7.5-20.4) and 37 (17%) men (IR=8.9/100 PY; 6.0-11.8). Women had more favourable profiles with respect to traditional risk factors for liver disease progression (younger, shorter duration of HCV infection and less alcohol use). Despite this, female sex was associated with a greater than two-fold increased risk of fibrosis progression (adjusted hazard rate (HR) =2.23; 1.22-4.08).

**Conclusions:**

HIV-HCV co-infected women receiving antiretroviral therapy were at significantly greater risk of progressing to liver fibrosis as measured by APRI compared with men. Enhanced efforts to engage and treat co-infected women for HCV are needed.

## Introduction

Due to shared routes of transmission, hepatitis C virus (HCV) and HIV co-infection is common. Approximately one quarter of HIV-infected Canadians are seropositive for HCV [1–2]. With improved outcomes on combination antiretroviral therapy (cART) AIDS related mortality has declined significantly, yet liver-related morbidity and mortality has progressively increased among co-infected patients [3–4] owing in part to more rapid hepatic fibrosis progression [3, 5–6]. Residual HIV-related immune dysfunction [7–8], and immune activation, cumulative cART induced hepatotoxicity [9] and metabolic changes are among the potential causes of accelerated liver disease progression [10].

Worldwide women represent a rapidly growing segment of the HIV infected population and in Canada account for 22% of all infections and 26% of new infections in 2010 [11]. Injection drug use (IDU) is an important route of HIV transmission in women [12]. Moreover, from 2006 to 2008, 15–24 year old women experienced the highest increase in rates of acute HCV infection, while men experienced stable or decreasing rates [13].

Sex has been shown to be an important predictor of chronic liver disease outcomes in HCV mono-infection, but few studies have assessed whether this holds true for co-infected individuals. Studies in HCV mono-infection have shown milder liver disease [14] and slower progression [15–18] in women compared to men. What accounts for these differences in the natural history of liver disease is not entirely clear and might relate to both socio-behavioral and biologic differences between the sexes. Similarly in HIV/HBV co-infection, male sex is associated with more advanced liver disease [19]. There is fairly consistent evidence from experimental and clinical studies in HCV [20–24] that such differences could be explained in part by the protective role of estrogens and/or estrogen receptors on fibrogenesis in premenopausal women. Postmenopausal status is characterized by accelerated fibrosis progression [25–26]. In HIV/HCV co-infection sex-based differences in cART associated liver toxicity and metabolic complications may further alter the risk of liver fibrosis progression [27].

The aim of our study was to investigate whether sex influences progression to liver fibrosis in ART treated HIV-HCV co-infected adults after accounting for socio-behavioural and clinical characteristics.

## Materials and Methods

### Study Population, Design, and Setting

We used data from the Canadian HIV-HCV Co-infection Cohort (CCC), a prospective multi-centre study recruiting patients 16 years of age or older with documented HIV infection (HIV-seropositive by enzyme-linked immunosorbant assay (ELISA) with western blot confirmation) and chronic HCV infection or evidence of HCV exposure (e.g. HCV seropositive by ELISA with RIBA II or EIA confirmation, and/or HCV RNA positive). One thousand one hundred and nineteen patients were enrolled from 17 sites across Canada from April 2003 to July 2012. Study details have been described elsewhere [4]. The CCC was approved by the community advisory committee of the CIHR-Canadian HIV Trials Network, the Biomedical B Research Ethics Board of the McGill University Health Centre (Protocol No. BMB-06-006t) the UBC-Providence Health Care Research Ethics Board (H08-00474), the Institutional Review Board Services, the Conjoint Health Research Ethics Board (20931), the Capital Health Research Ethics Board (CDHA-RS2007-118), the Windsor Regional Hospital Research Ethics Board (07-122-17), Veritas IRB, the Hamilton Integrated Research Ethics Board (06–397), the Comité d'éthique de la recherche du CHUM (2003–1582, SL 03.008-BSP), the Comité d'éthique de la recherche du CHU de Québec (C11-12-153), the Sunnybrook Health Sciences Centre Research Ethics Board (252–2008), the Health Sciences North Research Ethics Board (605), the University Health Network Research Ethics Board (06-0629-BE), the UBC-Providence Health Care Research Ethics Board (H08-00474), and the Ottawa Health Science Network Research Ethics Board (2007229-01H).

The analytic sample (n = 308) for this substudy we included participants chronically infected with HCV (defined as HCV RNA positive, lower detection limit: 50 IU/mL; Roche COBAS AMPLICOR assay, Roche Molecular Systems, Inc., Branchburg, NJ, USA), who were receiving cART at cohort entry and who had at least two consecutive visits (valid risk-set). The primary outcome of interest was progression to liver fibrosis; therefore, participants with significant fibrosis (see definition below) or radiological or histological diagnosis of cirrhosis and/or end-stage liver disease (ESLD) at study entry were excluded. Due to potential effects of HCV treatment on fibrosis progression, patients on HCV therapy at the time of enrolment were excluded. Patients were censored when they began HCV therapy during follow-up (because treatment can affect the AST and platelet counts which would impact the measurement of APRI score), when an outcome occurred, at death or on their last visit prior to July 2012.

### Data Collection and Outcome measures

All patients gave written informed consent before undergoing an initial evaluation and followed by study visits scheduled approximately every six months. At each visit, sociodemographic and behavioral information were self-reported by the patient in standardized questionnaires; medical treatments and diagnoses were collected by research personnel and blood was provided for laboratory assessments, including aspartate aminotransferase (AST) and platelet counts. The time since HIV diagnosis was calculated using the date of HIV seroconversion, if known, or the date of first HIV positive test. The duration of HCV was determined using the date of HCV seroconversion, if known, the year of first IDU as a proxy of HCV infection [28] or the date of first HCV positive test. Alcohol abuse was defined as self-reported alcohol intake of more than 2 drinks per day or binge drinking (greater than six drinks on one occasion at least once a month).

We used the AST-to-platelet ratio index (APRI) as a surrogate biomarker for liver fibrosis [29–30]. While liver biopsy is considered to be the gold standard for measuring fibrosis it is also invasive, costly, difficult to repeat, and therefore often results in limited sample size and selection bias when studying liver fibrosis. In addition, results may be affected by tissue sampling and interpretation error [31]. Transient elastography (Fibroscan) was not yet available. At each visit, the APRI was calculated as: [100 x (AST (U/L)/upper limit of normal)]/ platelet count (10^9^/L) [29]. Significant fibrosis was defined as an APRI score ≥ 1.5 corresponding to a liver biopsy score ≥F2 [29]. FIB-4 was calculated as follows: (age [yr] x AST [U/L]) / (PLT [10(9)/L]x (ALT [U/L])(1/2)). FIB-4>3.25 corresponds to a liver biopsy score of ≥F3. [32]

End-stage liver disease (ESLD) included documented diagnosis of liver cirrhosis, ascites, hepatic encephalopathy, bleeding esophageal varices, and spontaneous bacterial peritonitis captured using specific case report forms. Outcome measures focused on the incidence of developing an APRI score ≥1.5 and ESLD during follow-up.

### Statistical Analyses

We compared baseline characteristics of participants by sexes using Kruskall-Wallis test for continuous variables and Pearson’s χ^2^ or Fisher's exact tests for categorical variables. Tests were two-tailed and with a significance level of α = 0.05.

We estimated the incidence rates of developing an APRI≥1.5 by dividing the number of participants reaching the outcome by the number of person-years at risk, and expressed in cases per 100 person-years (P-Y) stratified by sex. Poisson count models were used to calculate confidence intervals (CI) for incidence rates. Kaplan-Meier plots for time to development of liver fibrosis were calculated comparing sexes.

Multivariate proportional hazards models were built to assess the effect of sex on progression to an APRI score ≥1.5 during follow-up and included covariates that were determined *a priori* to be clinically important. Because liver fibrosis was only assessed at each cohort visit but might have occurred at any point in the preceding interval (risk-set), we used a discrete time version of the Cox proportional hazards model with a complementary log log link function, an offset to allow for variation in the time between visits and robust standard errors [33] to account for repeated measures within individuals. The final multivariate model included female sex, baseline age, alcohol consumption, cigarette smoking, HCV duration, APRI and time-updated CD4 cell count and HIV viral load. The natural logarithm of the APRI [ln(APRI)], which nearly normalizes the distribution, was used in all analyses [34]. In sensitivity analysis we substituted FIB-4 > 3.25 for APRI as an alternate surrogate marker of fibrosis.

Statistical analyses were performed using Stata SE version 11 (Stata Corporation, College Station, Texas).

## Results

### Patient characteristics

**Fig 1:**
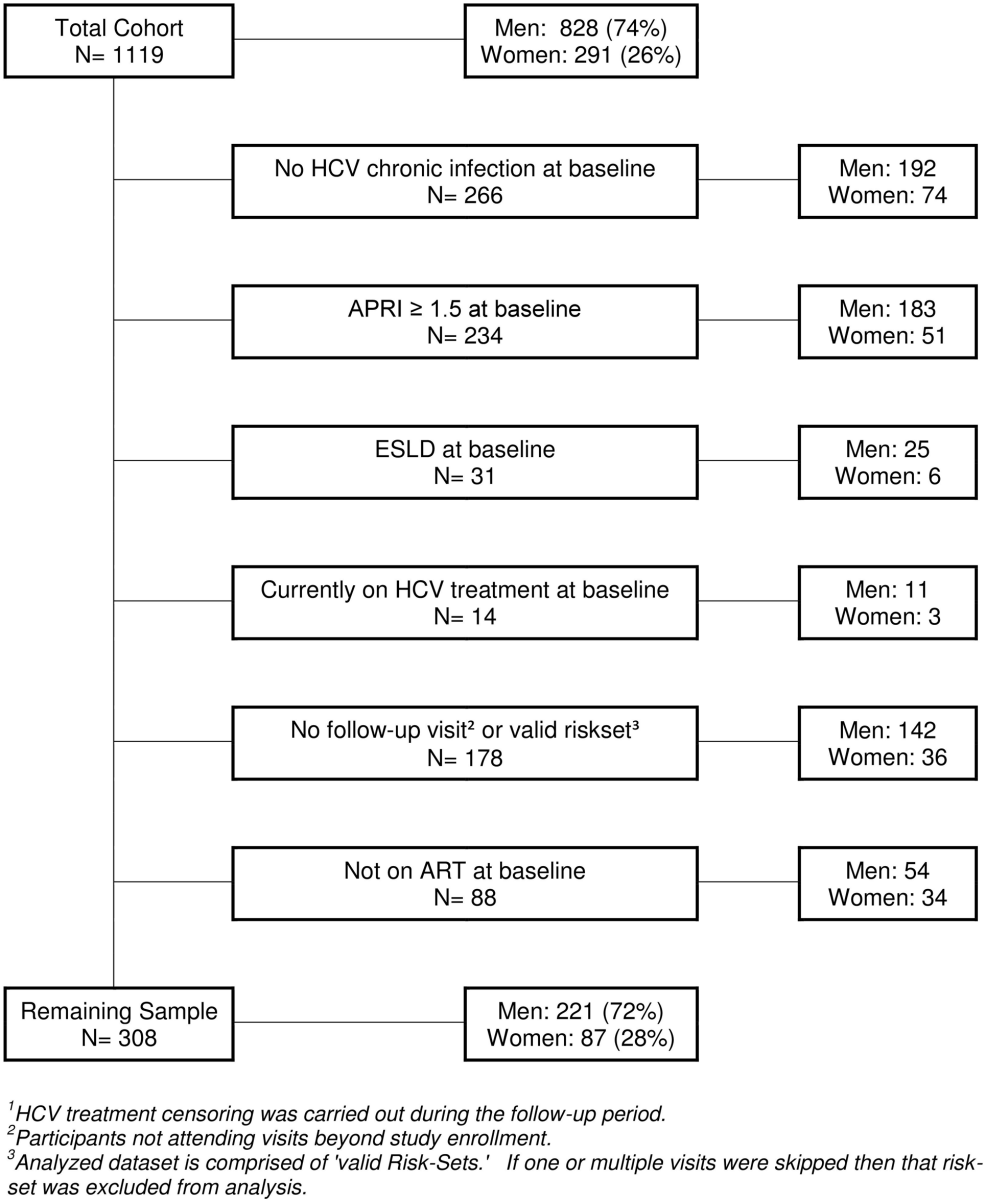
Participant Selection Flow Chart.

Of the 1119 patients enrolled in the CCC, a total of 308 patients satisfied the inclusion criteria (Fig 1), 87 (28%) of whom were women. The principal reasons for exclusion were negative/unknown HCV RNA status at baseline (n = 266) and presence of fibrosis or ESLD at baseline (n = 265) which did not differ between men and women. Included patients were similar to those excluded with the following exceptions: those included were more likely to be heterosexuals (80 vs. 71%), have suppressed HIV RNA (73 vs. 55%), be HCV genotype 1 or 4 (81 vs. 73%) and be naïve to HCV treatment (90 vs. 80%). Nadir CD4 and baseline APRI were higher among those excluded. Thirty-six (12%) participants were censored after initiating HCV therapy during follow-up; 10 (11%) women and 26 (12%) men (both had similar average APRI at time of treatment), while 69 (22% overall; 22% of women and 23% of men) participants were lost to follow-up (defined as having no study visit for at least 1.5 years).

Baseline demographic and clinical characteristics at enrolment by sex are presented in Table 1. At baseline women had a more favourable profile than men with respect to traditional factors associated with liver disease progression with shorter median duration of HIV and HCV infections, lower rates of alcohol abuse and younger age. However, women were more frequently Aboriginal, IDU, obese, and HCV treatment naive. CD4 cell counts, HIV RNA, type of cART regimen and HCV genotypes were similar in both men and women. Sixteen (5%) deaths occurred during follow-up; 10 (5%) in men and 6 (7%) in women; the majority were from drug overdose (n = 6), ESLD (n = 2) and cancer-related causes (n = 2).

**Table 1:** Sociodemographic and clinical characteristics of HIV-HCV co-infected patients at baseline by sex (2003–2012).

	TOTAL (N = 308)	Female (N = 87)	Male (N = 221)	p-values
Age (years), median (IQR)	44 (39, 50)	42 (37, 47)	45 (41, 50)	<0.01
Age ≥45 years, No. (%)	138 (45)	31 (36)	107 (48)	0.06
Aboriginal, No. (%)	48 (16)	22 (25)	26 (12)	<0.01
Greater than high school education, No. (%)	76 (25)	19 (22)	57 (26)	0.56
Gross monthly income >$1500, No. (%)	81 (26)	20 (23)	61 (28)	0.49
Heterosexual, No. (%)	246 (80)	81 (93)	165 (75)	<0.01
History of IDU, No. (%)	256 (83)	73 (84)	183 (83)	1.00
Active IDU, No. (%)	118 (38)	37 (43)	81 (37)	0.41
Current alcohol use, No. (%)	144 (47)	35 (40)	109 (49)	0.19
Current alcohol abuse, No. (%)	44 (31)	5 (14)	39 (37)	<0.05
Current cigarette smoking, No. (%)	235 (77)	70 (81)	165 (76)	0.36
Time since HIV diagnosis (years), median (IQR)	11 (7, 17)	10 (6, 15)	12 (8, 17)	0.16
Duration HCV infection (years), median (IQR)	19 (11, 26)	18 (11, 22)	20 (11, 27)	0.07
Nadir CD4 (cells/μL), median (IQR)	140 (50, 230)	140 (49, 249)	136 (57, 230)	0.99
CD4 count (cells/μL), median (IQR)	373 (250, 520)	380 (270, 520)	360 (240, 518)	0.60
HIVRNA load (log10 copies/mL), median (IQR)	1.7 (1.7, 1.8)	1.7 (1.6, 1.9)	1.7 (1.7, 1.8)	0.09
HIVRNA load ≤50 copies/mL, No. (%)	220 (73)	62 (72)	158 (73)	0.92
APRI, median (IQR)	0.5 (0.4, 0.8)	0.5 (0.4, 0.7)	0.5 (0.4, 0.8)	0.14
Time since first start of ART (years), median (IQR)	6 (2, 10)	5 (2, 10)	6 (2, 11)	0.75
cART regiment^a^, No. (%)				
PI	205 (67)	56 (64)	149 (67)	0.71
NNRTI	93 (30)	28 (32)	65 (29)	0.73
Others	16 (5)	7 (8)	9 (4)	0.16
HCVRNA load (log10 copies/mL)^b^, median (IQR)	6.2 (5.4, 6.7)	6.1 (5.4, 6.7)	6.2 (5.4, 6.8)	0.93
HCV treatment naïve, No. (%)	277 (90)	83 (95)	194 (88)	0.06
HCV genotype (1 & 4), No. (%)	210 (81)	58 (83)	152 (81)	0.85
Chronic Hepatitis B (HBsAg positive), No. (%)	12 (5)	2 (3)	10 (6)	0.52
Obese (BMI ≥30), No. (%)	31 (11)	14 (18)	17 (9)	<0.05

Abbreviations: HCV, hepatitis C virus; HIV, human immunodeficiency virus; IDU, injection drug use;

APRI, aspartate aminotransferase to platelet ratio; AST, aspartate aminotransferase; BMI, body mass index;

cART, combination antiretroviral; PI, protease inhibitor; NNRTI, non-nucleoside reverse transcriptase inhibitor.

^a^Sum of regimens >100% as some participants are on both PI, NNRTI and/or other cART.

^b^For HCVRNA VL only 166 (34/87 (39%) female and 132/221 (60%) male) had available quantitative HCV RNA values.

### Progression to liver fibrosis and ESLD

The median (inter-quartile range (IQR)) follow up time at risk (including valid risk-sets only) was 1.4 years (0.7–2.5) overall; 1.5 (0.7–2.6) years in women and 1.3 (0.8–2.0) years in men. The cumulative follow-up time at risk was 544 person-years (129 person-years in women and 415 person-years in men).

**Fig 2:**
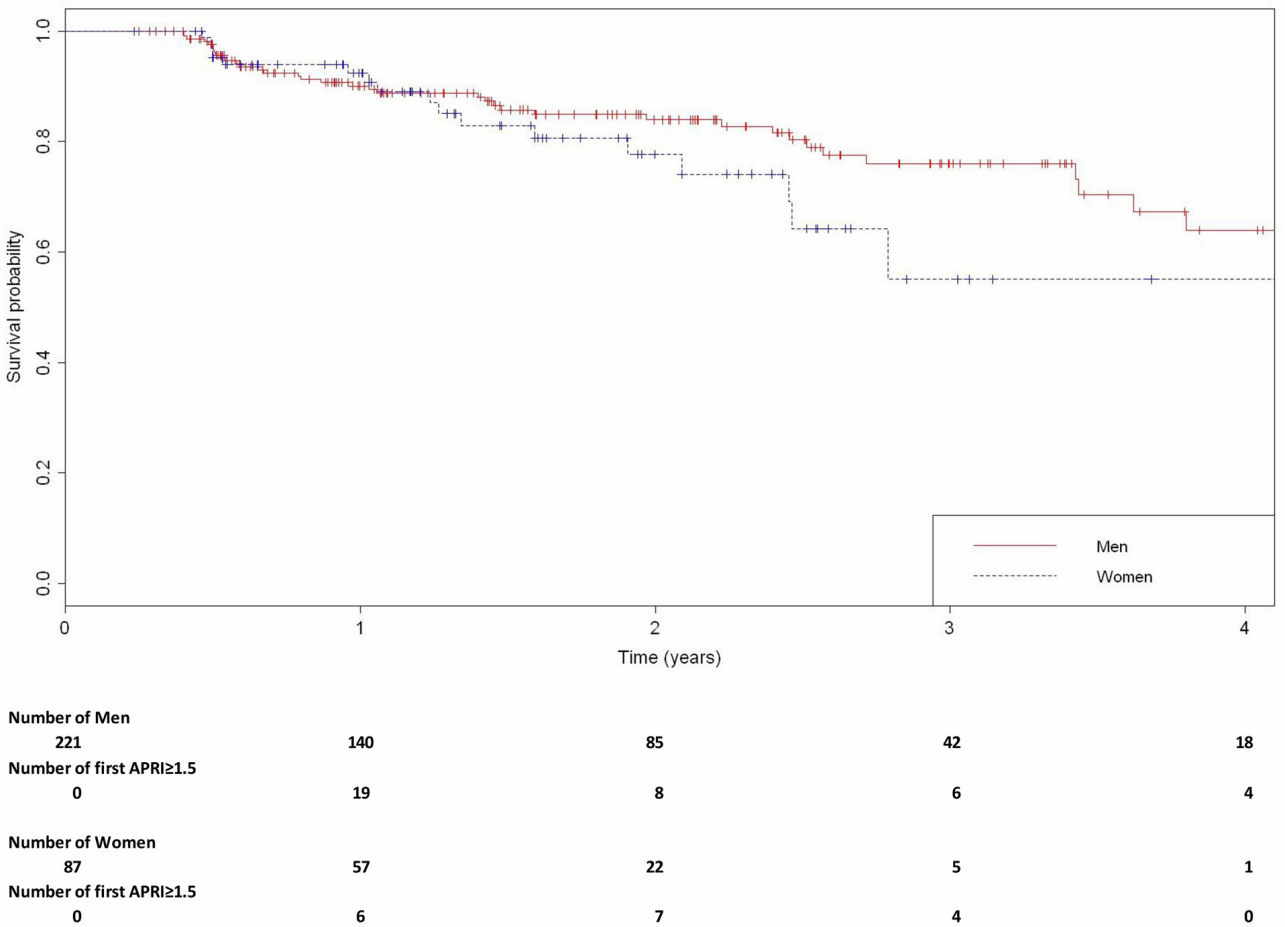
Kaplan Meier time to significant fibrosis stratified by sex.

A total of 55 (18%) participants developed an APRI score ≥ 1.5 (10.1/100 person-years; 95% CI, 7.4–12.8); 18 (21%) were women (14.0/100 person-years; 95% CI, 7.5–20.4) and 37 (17%) were men (8.9/100 person-years; 95% CI, 6.0–11.8). The survival probability of developing hepatic fibrosis over time stratified by gender is shown in Fig 2 (log rank: p = 0.12).

Among patients who progressed to an APRI ≥1.5, HCV duration was longer and baseline APRI was significantly higher than in non-progressors. Progressors were: more often women, older, heterosexual, less educated, current smokers, current alcohol users, and HCV treatment experienced as compared to non-progressors. Among women, those who progressed to fibrosis had longer HCV duration and were more frequently of presumed menopausal age (defined as ≥45 years old) at time of progression (61 vs. 42%, p = 0.19) than women who did not progress. The only notable differences between women of menopausal age and younger women at study entry were the longer HCV infection duration and greater alcohol consumption in women of menopausal age; both factors associated with accelerated liver disease. Data on past pregnancy, hormonal contraceptive use and hormone replacement therapy (HRT) were not available.

**Fig 3:**
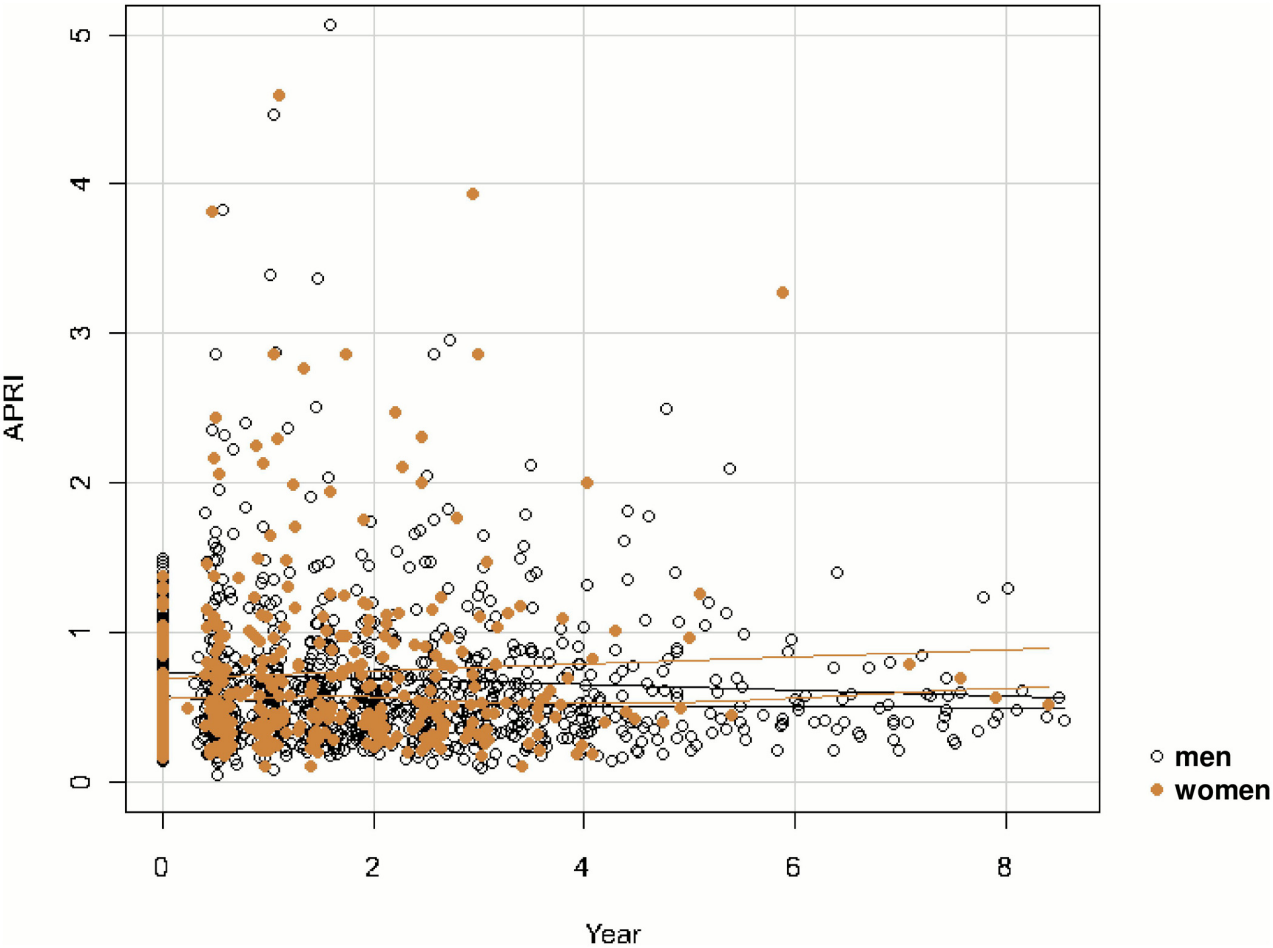
APRI over time for men and women. Note: All available APRI measures for the study population are shown (e.g. not restricted to measures contained within the risk intervals used for the discrete-time proportional hazards analysis).

Fig 3 show that the majority of people had relatively slow progression of APRI whereas a subset of both men and women appear to progress more rapidly, and that there may be more of these rapid progressors among women.

In univariate discrete time proportional hazard analyses (Table 2) female sex, baseline smoking and alcohol consumption, history of IDU and detectable HIV viral RNA were positively associated with fibrosis progression but only baseline APRI reached statistical significance.

**Table 2:** Discrete time proportional hazards models of factors associated with development of significant fibrosis (APRI ≥ 1.5) in follow-up.

	Univariate models		Multivariate model	
	Unadjusted HR	95% CI	Adjusted HR	95% CI
**Time independent baseline covariates**				
Female sex	1.58	(0.91, 2.76)	2.23	(1.22, 4.08)
Age (years)	1.01	(0.98, 1.04)	1.01	(0.98, 1.05)
Age ≥45 years	1.61	(0.93, 2.77)	---	---
Aboriginal ethnicity	0.99	(0.44, 2.20)	---	---
History of IDU	1.67	(0.71, 3.89)	---	---
Current alcohol use	1.50	(0.87, 2.59)	1.52	(0.85, 2.70)
Current cigarette smoking	1.84	(0.86, 3.96)	1.47	(0.68, 3.19)
Duration HCV (per 5 years)	1.10	(0.97, 1.25)	1.11	(0.96, 1.29)
cART regiment				
PI	1.63	(0.88, 3.02)	---	---
NNRTI	0.72	(0.38, 1.36)	---	---
Others	0.63	(0.15, 2.58)	---	---
ln (APRI)	4.26	(2.15, 8.42)	4.71	(2.38, 9.31)
Obese (BMI ≥30)	1.02	(0.46, 2.26)	---	---
**Time updated covariates**				
CD4 count (per 100 cells/μL)	1.04	(0.92, 1.18)	1.05	(0.95, 1.17)
HIVRNA load (per log10 copies/mL)	1.24	(0.94, 1.62)	1.32	(0.97, 1.80)

Abbreviations: IDU, injection drug use; HCV, hepatitis C virus; cART, combination antiretroviral; PI, protease inhibitor;

NNRTI, non-nucleoside reverse transcriptase inhibitor; APRI, aspartate aminotransferase to platelet ratio;

BMI, body mass index; HR, hazard ratio; CI, confidence interval.

Female sex was significantly associated with fibrosis progression after adjustment in the multivariate model (aHR 2.23; 95% CI: 1.22–4.08). Other factors positively associated with developing an APRI score ≥ 1.5 during follow-up were baseline ln(APRI) (aHR 4.71; 95% CI: 2.38–9.31), baseline alcohol use (aHR 1.52; 95% CI: 0.85–2.70), cigarette smoking (aHR 1.47; 95% CI: 0.68–3.19) and time-updated HIV RNA (aHR 1.32; 95% CI: 0.97–1.80 per log10 copies/mL). In other adjusted models, the effect of Aboriginal ethnicity, IDU, cART type, chronic HBV co-infection, HCV genotype and obesity (BMI ≥30) were examined and none was found to be associated with fibrosis progression nor did their inclusion in the models alter the main results (data not shown).

Given that menopause in women may play a role in fibrosis progression, we performed a *post hoc* sensitivity analyses where age was dichotomized at ≥45 years (as a surrogate for menopausal status), with an interaction term between age ≥45 years and sex. The effect of female sex was increased (aHR 2.34, 95% CI: 1.04–5.34) but the interaction term was not significant (aHR 0.96, 95% CI: 0.31–3.04). Since women may be more vulnerable to the effect of alcohol due to their smaller volumes of distribution and restricted alcohol dehydrogenase activity [35], we performed a second *post hoc* sensitivity analyses that included an interaction term between female sex and alcohol consumption at baseline. The interaction term was not significant (aHR 0.54, 95% CI: 0.17–1.70) but its inclusion in the model amplified the estimates for both female sex (aHR 3.09, 95% CI: 1.28, 7.43) and alcohol consumption (aHR 1.89, 95% CI: 0.89–4.04).

Finally, we repeated the main model replacing the APRI with FIB-4 >3.25 and results were similar (aHR for female sex: 1.79 95% CI 0.83–3.5).

Overall, development of ESLD during follow-up was observed in 13 (5%) participants (2.1/100 person-years; 95% CI, 1.1–3.7); 2 women (1.3/100 person-years; 95% CI, 0.2–4.7) and 11 men (2.4/100 person-years; 95% CI, 1.2–4.3). The baseline mean duration of HCV infection for participants who had progressed to ESLD was 15.9 years (IQR: 13.9–18.0) for women and 19.3 years (IQR: 9.8–21.4) for men. The specific diagnoses were: 2 cirrhosis, 2 ascites, 3 hepatic encephalopathy, 1 bleeding esophageal varices, 1 portal hypertension and 4 hepatocellular carcinoma.

## Discussion

Contrary to what has been observed in HCV mono-infection, our study found cART treated co-infected women to be at significantly greater risk of progressing to liver fibrosis, as measured by APRI score, compared to men even after adjusting for well-established potential confounders. A high proportion of women, typically under-represented in randomized controlled trials, were present in this prospective cohort of HIV/HCV co-infection. Over a relatively short period of follow-up we found a moderate rate of fibrosis progression with 18% of patients reaching an APRI score ≥1.5 and 5% developing end-stage liver disease overall. Women were over two times more likely to develop fibrosis despite having fewer traditional risk factors for liver disease progression at baseline. Incidence of clinical ESLD events was similar between men and women but we were underpowered to detect a difference in rates between the sexes. However, the median time from HCV infection to ESLD was shorter in women suggesting that the fibrosis progression rates observed might translate into worse clinical outcomes in the long term.

Ours is the first longitudinal study to specifically examine the effect of biological sex on liver fibrosis progression in HIV-HCV co-infected patients. Two studies in co-infected patients are supportive as they also found female sex to be independently associated with fibrosis progression or hepatic decompensation with effect sizes very similar to those we observed, although little was made of these findings. In a cross sectional analysis, Mehta et al [36] evaluated determinants of liver fibrosis among 210 HIV/HCV co-infected patients on cART at a single clinic undergoing liver biopsy. Female sex was associated with both bridging fibrosis/cirrhosis and necroinflammatory activity (OR = 2.8, 95% CI: 1.4–5.8 and OR = 3.5, 1.8–7.5, respectively). In a longitudinal study, Pineda et al [37] followed a cohort of 1011 co-infected patients in Spain including 24% women. They reported an association between female sex and hepatic decompensation (HR = 2.11, 95% CI: 1.07–4.15). A recent study of 435 liver biopsy pairs in HIV/HCV coinfected patients showed women to be at elevated risk of fibrosis progression (OR from 1.08 to 1.25) although this association was not found to be statistically significant [38]. In contrast, in serial biopsy-based [5, 39–40] and other cross-sectional studies [6, 41], no effect of sex was found on estimated moderate or advanced liver fibrosis progression rates in co-infection. It is possible that the lower proportion of women enrolled in these latter studies may have precluded finding sex-specific effects.

The reasons co-infected women may differ from HCV mono-infected women with respect to risk of fibrosis progression are unclear. Metabolic complications of cART or cART induced hepatotoxicity may differ between men and women [27, 42–43] and impact liver fibrosis in women [44]. In our study, the type of current ART was not associated with fibrosis progression and we did not observe differences in cART regimens used between sexes. However, we did not evaluate drug-specific cumulative exposures. It has also been reported that women may be more likely to interrupt ART compared to men [45] and in previous work we have demonstrated that ART interruption is associated with fibrosis progression [46].

Socio-demographic and risk behaviour patterns differed substantially between the sexes. In general the women in this cohort had more favourable profiles with respect to traditional risk factors for liver disease progression such as age, duration of HCV infection and alcohol exposure. Despite this, rates of hepatic fibrosis progression among women remained elevated after adjusting for these factors. Although women are believed to be more susceptible to lower levels of alcohol exposure, our interaction model did not show a significant relationship between liver fibrosis progression and female sex by alcohol consumption status.

A plausible explanation for differential rates of fibrosis progression in co-infected women may be the protective effect of estrogens on the liver through the inhibition of stellate cell proliferation [22, 25] which is attenuated after menopause. Clinically, several HCV mono-infection studies [23–26] have shown that with the onset of menopause, liver fibrosis progression markedly increases in women and the response to HCV therapy with pegylated interferon/ ribavirin is decreased [10]. After menopause women change from an estrogen protective milieu, where HCV-mediated inflammation is controlled, to a hepatic proinflammatory state where estrogen levels drop and IL-6 and TNF-α cytokines levels greatly increase possibly increasing the risk of hepatic fibrosis progression. In HIV-positive women amenorrhea, irrespective of age, and/or early onset of menopause may be more common than in HIV uninfected women [47–48] due to illicit substances use, psychotherapeutic medications [49] and low CD4 cell count [50] potentially exposing them to longer periods of estrogen depletion. Amenorrhea is also an extrahepatic manifestation of chronic hepatitis infection [51] which could further compound the risk of disease progression [10]. Our study failed to detect a significant difference in developing liver fibrosis between women of presumed menopausal age and younger women though a higher proportion of women who progressed were of menopausal age suggesting we may have been underpowered. Our categorization of menopausal status however was rudimentary (age >45 years) given that menopausal age varies from one study to another [10, 50, 51] and we lacked specific data on menstrual histories and sex hormone levels. The median age of women was 42 years at baseline consistent with premature menopause/amenorrhea if estrogen depletion is what accounted for the greater risk of fibrosis among women in our study.

The present study has notable strengths, in particular its high representation of women, diversity in patient’ risk profiles, enrollment from a broad geographic area and a variety of models of care and practice settings. It is also subject to potential limitations. The analysis was limited to patients who attended clinic visits, thus our findings may not be generalizable to the wider HIV-HCV co-infected population not actively engaged in medical care. We used APRI score as a surrogate marker for liver fibrosis instead of liver biopsy for reasons previously stated. Multiple studies have used the APRI score and demonstrated its value in predicting liver fibrosis progression, hepatic decompensation and mortality including among women [34, 46, 52–53]. The APRI score has been shown to perform similarly to other markers of liver fibrosis including FIB-4 [30]. The APRI score lacks sensitivity and may misclassify individuals as not having fibrosis, however is highly specific for fibrosis stage ≥F2 (0.93, 95% CI: 0.91, 0.94) [54]. Thus using APRI ≥1.5 as an outcome is conservative. While we may have underestimated the degree of fibrosis present among those with scores below 1.5, any such under-ascertainment is unlikely to vary by sex. APRI measurement is subject to fluctuations in AST and platelet levels due to other factors not necessarily associated with liver fibrosis progression. There is however no evidence that in co-infection, women experience greater increases in AST after ART initiation compared with men that would bias our results [55]. Furthermore, true “normal” values for AST are lower for women than for men. In calculating the APRI score, we applied the same laboratory defined upper limit of normal to both sexes which would tend to underestimate fibrosis in women as compared with men. To account for potential gender related differences in estimating fibrosis, we adjusted for baseline APRI in our models. The use of the dichotomous outcome of APRI >1.5 was chosen because it represents a clinically meaningful cut-off (e.g. both an indication for treatment and is prognostic of poor outcomes such as death and liver failure). Such an outcome may mask non-linear or heterogeneous progression rates. It is possible that there is a subset of women that are more rapid progressors and account for the associations we observed.

The paucity of information on obstetric, gynecological and contraceptive history did not enable us to investigate the effect of those exposures on liver disease. Finally, as our methods required two consecutive follow-up visits (to form discrete at-risk intervals of 6 months) it is possible that we could have introduced a ‘healthy women effect’. Although we did not observe a difference in losses to follow-up by sex, if healthier women were more prone to miss visits for childcare or work related responsibilities, their observed follow-up time at risk would be decreased which may exaggerate the estimates of fibrosis progression among women since sicker women would be over-represented in the analyses, in contrast to the `healthy worker effect’ where the sicker workers tend to leave the workforce.

## Conclusions

Among HIV-HCV co-infected patients receiving ART, women were at significantly greater risk of progressing to liver fibrosis as measured by APRI compared to men. Further prospective studies and long term follow-up of co-infected patients are needed to assess the effects of potential interactions between sex and cumulative antiretroviral exposure on progression of liver diseases and better clarify the underlying sex-specific mechanism that could explain increased risk of liver fibrosis among women. The ideal approach to reduce progression to liver fibrosis in HIV-HCV co-infected patients is by curing HCV through HCV therapy. Given the lower rates of treatment initiation among women we observed, enhanced efforts to engage and treat co-infected women for HCV are needed.
